# *Carica papaya* Reduces Muscle Insulin Resistance via IR/GLUT4 Mediated Signaling Mechanisms in High Fat Diet and Streptozotocin-Induced Type-2 Diabetic Rats

**DOI:** 10.3390/antiox11102081

**Published:** 2022-10-21

**Authors:** Jeane Rebecca Roy, Coimbatore Sadagopan Janaki, Selvaraj Jayaraman, Vijayalakshmi Periyasamy, Thotakura Balaji, Madhavan Vijayamalathi, Vishnu Priya Veeraraghavan

**Affiliations:** 1 Department of Anatomy, Bhaarath Medical College and Hospital, Bharath Institute of Higher Education and Research (BIHER), Chennai 600 073, Tamil Nadu, India; 2 Centre of Molecular Medicine and Diagnostics (COMManD), Department of Biochemistry, Saveetha Dental College & Hospitals, Saveetha Institute of Medical & Technical Sciences, Saveetha University, Chennai 600 077, Tamil Nadu, India; 3 Department of Biotechnology and Bioinformatics, Holy Cross College, Trichy 620 002, Tamil Nadu, India; 4 Department of Anatomy, Chettinad Hospital and Research Institute, Chettinad Academy of Research and Education, Chennai 603 103, Tamil Nadu, India; 5 Department of Physiology, Bhaarath Medical College and Hospital, Bharath Institute of Higher Education and Research (BIHER), Chennai 600 073, Tamil Nadu, India

**Keywords:** *Carica papaya*, insulin resistance, diabetes mellitus, skeletal muscle, insulin receptor, glucose transporter 4, molecular dynamics, therapeutic implications

## Abstract

In the management of type 2 diabetes, oral antidiabetic drugs have several side effects, which in turn have led the pharmaceutical industry to search for good therapeutic, non-toxic and reliable drugs. *Carica papaya (C. papaya)* is one of several plants in nature that have been found to possess anti-diabetic properties. Despite studies being focused on the antidiabetic activity of *C. papaya*, the molecular mechanism against high fat diet induced insulin resistance is yet to be identified. The role of *C. papaya* was evaluated on insulin signaling molecules, such as the insulin receptor (IR) and glucose transporter-4 (GLUT4) in high fat, diet-streptozotocin induced type 2 diabetic rats, and analyzed the bioactive compounds of *C. papaya* against IR and GLUT4 via molecular docking and dynamics. The ethanolic extract of *C. papaya* leaves (600 mg/kg of body weight) was given daily to male wistar rats for 45 days and we observed the various biochemical parameters, gene expression analysis and histopathology of skeletal muscle. Molecular docking and dynamics were undertaken to understand the bioactive compounds with the greatest hit rate. *C. papaya* treatment was able to control blood glucose levels, the lipid profile and serum insulin, but it facilitated tissue antioxidant enzymes and IR and GLUT4 levels. The in-silico study showed that kaempferol, quercitin and transferulic acid were the top three ligands with the greatest hit rate against the protein targets. Our preliminary findings, for the first time, showed that *C. papaya* reinstates the glycemic effect in the diabetic skeletal muscle by accelerating the expression of IR and GLUT4.

## 1. Introduction

Diabetes mellitus has become a global and pressing concern in public health. It is a conspicuous, non-transmissible disease that poses a serious threat to world health. However, the fastest growing regions for diabetes in the future are Asia, the Middle east and the Africa, where diabetes is projected to rise by 50% by 2030 [1]. Diabetes mellitus is a circuitous interaction of genetic, environmental and demographic influences, and is marked by hyperglycemia, which becomes worse with time and causes disruptions in carbohydrate, protein and lipid metabolism [2]. These progressively lead to vision loss, renal disorders, cardio- and cerebro-vascular diseases [3]. Static lifestyles, coupled with increased urban sprawl habits and processed food, show the prevalence of diabetes mellitus will be three-fold higher in the next 25 years and this also involves young populations [4].

In type 2 diabetes, individuals develop insulin resistance in the peripheral target tissues, thereby prompting a high demand for insulin from the overexerted beta cells of the pancreas. A declination in insulin secretion was observed with increased insulin demand over time due to progressive cell death and the majority of type 2 diabetes patients were not reliant on insulin when insulin secretion continued and insulin depletion seldom occurred [5]. A high fat diet can lead to the formation of excessive reactive oxygen species (ROS) that consequently lead to increased β-oxidation. This can affect the normal regulation of glucose and lipid metabolism [6]. Inflammatory cytokines and insulin signaling genes are altered, thereby disrupting the insulin signaling cascade. Intra-muscular lipids may build when the rate of β-oxidation outpaces the rate of fatty acid absorption, which may have negative consequences for the insulin action, which imparts insulin resistance [7]. This may lower the amount of sarcolemmal glucose transporter 4 (GLUT4), thus preventing glucose from entering the muscle and preventing glycolysis, glucose oxidation, and glycogen synthesis [8]. The “Randle Cycle” refers to the phenomenon whereby glucose oxidation decreases while fatty acid oxidation increases [9]. The general consensus is that reducing muscle fatty acid absorption or its esterification into other lipid intermediates will improve insulin sensitivity and prevent the negative consequences of lipid buildup in the myocyte [8].

Several plants have been found to possess anti-diabetic properties. Among them, *Carica papaya* (*C. papaya*) stands out in the list. The different parts of *C. papaya* have been used for many years because of its therapeutic applications. Previous literature has reveal the strong medicinal properties of *C. papaya*, namely anti-bacterial, anti-viral, anti-oxidant, hypoglycemic and anti-inflammatory activities [10]. Numerous studies have focused on the anti-diabetic activity of leaves, fruits and seeds of *C. papaya,* but the molecular mechanism is largely unknown. In this study, we focused on the effect of *C. papaya* leaves on the insulin signaling molecules, such as the insulin receptor and GLUT-4, as well as molecular docking and the simulation analysis of the bioactive compounds of *C. papaya* against IR and GLUT-4 to prove its mechanisms of action. Therefore, our research was executed in vivo and in silico to understand the role of *C. papaya* in insulin signaling and gene expression analysis in the skeletal muscles of a high fat diet and streptozotocin-induced type-2 diabetic experimental rats.

## 2. Materials and Methods

### 2.1. Chemicals and Reagents

The chemicals and reagents employed in this study were obtained from Sigma Chemical Company, St. Louis, MO, USA; Crystal Chem Inc., Elk Grove Village, IL, USA; MP Biomedicals, Santa Ana, CA, USA; Invitrogen, Waltham, MA, USA; New England Biolabs (NEB), Ipswich, MA, USA; Promega, Madison, WI, USA; Eurofins Genomics India Pvt Ltd., Bangalore, India; KRISHGEN Bio-Systems, Worli, Mumbai, India and Abbkine Scientific Co, Ltd., Wuhan, China. On-Call Plus Blood glucose test strips were from ACON Laboratories, Inc., San Diego, CA, USA. An ultra-sensitive rat insulin enzyme-linked immunosorbent assay (ELISA) kit was acquired from Crystal Chem Inc. The total RNA isolation reagent (TRIR) was acquired from Invitrogen. The reverse-transcriptase enzyme (MMuLv) was procured from New England Biolabs (NEB), and the Go Taq Green master mix was bought from Promega. The insulin receptor (IR), glucose transporter-4 (GLUT4) and β-actin primers were obtained from Eurofins Genomics India Pvt Ltd., Bangalore, India. The serum insulin kit was acquired from KRISHGEN BioSystems, Worli, Mumbai MAH 400018, India. Kits for enzymatic antioxidants, oxidative stress markers and lipid profile were procured from Abbkine Scientific Co, Ltd., Wuhan, China.

### 2.2. Collection of Plant Material

The *C. papaya* leaves were collected from Kerala. They were shade dried and powdered. The material was authenticated by the National Institute of Siddha, Chennai, India. Certificate No: NISMB4392020.

### 2.3. Phytochemical Qualitative Analysis of C. papaya

The ethanolic extract of *C. papaya* was prepared using a vacuum rotary evaporator and was used for the phytochemical analysis. The presence of phytosterols, triterpenoids, flavonoids, phenols, tannin, alkaloids, saponin, acid, proteins, carbohydrates and glycosides were analyzed using the standard methods [11].

### 2.4. In Vitro Antioxidant Analysis

#### 2.4.1. DPPH Radical Scavenging Activity of *C. papaya*

Scavenging of the 2,2-Diphenyl-1-picrylhydrazyl (DPPH) radical was assessed using the method developed by Hatano et al. [12]. 1 mL of the DPPH solution was added to 1 mL of *C. papaya* at various concentrations (100–500 µL). The action was assessed at 517 nm after 50 min incubation. Ascorbic acid was taken as standard.

DPPH radical scavenged (%) = Control OD-Sample OD × 100 (1)

Control OD(2)

#### 2.4.2. Nitric Oxide Radical Scavenging Activity of *C. papaya*

Scavenging of the nitric oxide radical was assessed using the method developed by Garrat et al. [13]. 2 mL of 10 mM sodium nitroprusside in a 0.5 mL phosphate buffer saline (pH 7.4) was added to 0.5 mL of *C. papaya* at 100–500 µL concentrations and was incubated at 25 °C for 150 min. 0.5 mL of the mixture was added to 1.0 mL of sulfanilic acid reagent. Subsequently, 1.0 mL naphthylethylenediamine dihydrochloride (0.1% *w*/*v*) was added and kept for half an hour. The activity was determined at 540 nm. The scavenging activity was calculated as:

Inhibition% = A_0_ − A_1_ × 100 (3)

A_0_ (4)

#### 2.4.3. Superoxide Anion Scavenging Activity of *C. papaya*

Scavenging of super oxide anion activity was assessed using the approach developed by Liu et al. [14]. 3 mL of tris-HCl buffer was made with 0.75 mL nitroblue tetrazolium, 0.75 mL NADH solution and 0.3 mL of 100–500 µL concentrations of the *C. papaya*. 0.75 mL PMS was supplemented in the mixture. The absorbance at 560 nm was assessed using a spectrophotometer after 5 min incubation. The superoxide anion scavenging activity was calculated as follows:

Inhibition% = A_0_ − A_1_ × 100 (5)

A0(6)

### 2.5. In Vivo Study

#### Animals

Adult male Wistar albino rats (150–180 days old) were maintained under standard environ-mental conditions with a standard temperature (21 ± 2 °C), under specific humidity, and continual 12 h darkness and 12 h light cycles, as per the guidelines from the institutional animal ethical committee. The animals were maintained under a sterilized paddy husk as a bedding material in poly-propylene cages and were fed with standard pellets and water ad libitum at the Central Animal House, Saveetha Dental College, Chennai, Tamil Nadu, India. The research was permitted by the institutional animal ethical committee (IAEC No: BRULAC/SDCH/SIMATS/IAEC/08-2021/071 dated 21 August 2021).

### 2.6. Induction of T2DM

A high fat diet HFD (66 percent typical rat feed and 3 percent cholesterol, 1 percent cholic acid and 30 percent coconut oil) was catered to the rats for 4 weeks. After 4 weeks with high fat diet (HFD) feeding, the rats were injected intraperitoneally with a low dose of streptozotocin (STZ) (35 mg/kg) (Sigma Aldrich, St. Louis, MO, USA) [15]. For the following two days of STZ injection, the rats with a fasting blood glucose level (>120 mg/dL) were considered for the experiment. Diabetic rats were allowed to feed on HFD and sucrose water during the study.

### 2.7. Experimental Design

The rats were randomly divided into 5 groups of 8 rats each.

Group 1—Control rats; Group 2—Diabetic rats; Group 3—Diabetic rats + 600 mg/kg bwt ethanolic extract of *C. papaya* for 45 days; Group 4—Diabetic rats + 50 mg/kg bwt of metformin for 45 days; and Group 5—Control + 600 mg/kg bwt ethanolic extract of *C. papaya* for 45 days.

Fasting blood glucose (FBG) and oral glucose tolerance tests (OGTT) were carried out for the groups 1–5 2 days prior to sacrifice. On the last day of the experiment, the animals were sedated with sodium thiopentone (40 mg/kg body weight) and blood was drawn through cardiac puncture. Sera was also separated and stored at −80 °C. The blood was removed from the organs by injecting 20 mL of isotonic sodium chloride solution via the left ventricle. The gastrocnemius muscle was dissected instantly, according to the following parameters.

### 2.8. FBG

FBG measurements were taken after overnight fasting via On Call Plus blood glucose test strips (ACON Laboratories Inc., San Diego, CA, USA). The blood was taken from the tip of the rat tail and the results were indicated as mg/dL.

### 2.9. OGTT

Glucose load (10 mL kg; 50% *w*/*v*) was administered orally to the overnight fasting animals and the blood glucose levels were estimated at three time periods (60,120 and 180 min) using On-Call Plus blood glucose test strips. The zero-minute value was measured as the FBG value and results were indicated as mg/dL.

### 2.10. Serum Insulin

Serum insulin was calculated using the Krishgen kit according to the manufacturer’s protocol.

### 2.11. Homeostasis Model Assessment for Insulin Resistance (HOMA-IR)

HOMA-IR was determined using the following formula: fasting blood glucose X fasting serum insulin/405, as per the approach developed by Matthews et al. [16].

### 2.12. Lipid Profile

The lipid profile markers, such as serum triglycerides (TG), total cholesterol (TC), high density lipoproteins (HDL) and low-density lipoproteins (LDL), were determined according to manufacturer’s protocol for the biochemical analyzer using the kit from Abbkine Scientific Co, Ltd., Wuhan, China.

### 2.13. Glycogen Level

Estimation of glycogen in the gastrocnemius muscles of all the five groups in this study were analyzed using the method developed by Hassid and Abraham [17].

### 2.14. Oxidation Stress Marker

The amount of LPO in the skeletal muscle of the rats in this study were evaluated using the kit from Abbkine Scientific Co, Ltd., Wuhan, China, as per the manufacturer’s protocol.

### 2.15. Enzymatic Antioxidants

Assessment of enzymatic antioxidant markers, such as superoxide dismutase (SOD), catalase (CAT) and glutathione peroxidase (GPx) in the skeletal muscle of the experimental and the control rats, were examined using the kit from Abbkine Scientific Co, Ltd., Wuhan, China.

### 2.16. mRNA Expression Analysis

#### Total RNA Isolation, cDNA Conversion and Real-Time PCR

By means of a TRIR kit (Total RNA Isolation Reagent Invitrogen), total RNA was separated from the five groups. The reverse transcriptase kit was obtained from Eurogentec (Seraing, Belgium). The cDNA was made from 2 micrograms of the total RNA. The sequence of the primers employed in this study is given in Table 1. The reference gene used was β-actin. The genes were amplified in the real time PCR system (Stratagene MX 3000P, Agilent Technologies, 530l, Stevens Creek Blvd, Santa Clara, CA, USA) under the following reaction conditions: Initial denaturation at 95 °C for 5 min followed by 40 cycles of 95 °C for 30 s, 59–60 °C for 30 s and 72 °C for 30 s. Relative quantification was derived from the melt and amplification curve analyses.

### 2.17. Histopathology

The histopathology of the gastrocnemius muscle was carried out in ten-percent neutral buffered formalin fixed in paraffin, sectioned, stained with hematoxylin and eosin dye [21]. Semi thin sections of 0.5–1 microns were obtained using the LKB ultra-microtome and were identified using Olympus light microscope fitted with a Nikon digital camera and taken with a magnification of ×200.

### 2.18. Statistical Analysis

To ascertain individual differences of using computer-based tools, the control and care groups, Duncan’s one-way ANOVA and multiple range tests were used to assess the experimental results (Graph Pad Prism Version 5). The values with *p* < 0.05 were considered statistically significant.

### 2.19. Molecular Docking

#### 2.19.1. Compound/Ligand Preparation

The structures of the selected phytoconstituents from *C. papaya* (Table 2) were obtained in the structural data format (SDF) from the PubChem database. These SDF files were then produced using the “prepare ligands” module from the DSBDS software and filtered using the “Filter by Lipinski Rules” module. This procedure eliminated duplicate entries, computed isomers and tautomers, and created and reduced 3D conformations. BIOVIA is a product of Dassault Systems. The ADMET screening performed by BIOVIA predicts human intestinal absorption (HIA) following oral treatment. A well-absorbed chemical is one that is absorbed into the bloodstream of humans at a rate of at least 90%. The intestinal absorption model provides confidence ellipses of 95% and 99% in the ADMET PSA 2D, ADMET AlogP98 plane. Lipinski’s rule of five was used to calculate drug similarity properties. This rule states that the absorption of an orally delivered substance is more credibly improved if the molecule meets no less than three of the following rules:

- Hydrogen bond donors (OH, NH and SH atoms) less than or equal to 5;
- Hydrogen bond acceptors (N, O, and S atoms) 10;
- Molecular weight 500;
- log P;
- Compounds that violate any of the above principles are dubious to own a high rate of oral bioavailability [22].

**Table 2:** List of selected compounds from *C. papaya*.

S.No	Compound Name
1	Caffeic acid
2	Chlorogenic acid
3	Kaempferol
4	Quercetin
5	Rutin
6	p-coumaric acid
7	Trans-Ferulic acid
8	Protocatechuic acid

The pharmacokinetic characteristics (ADMET) and compliance with Lipinski’s rule of five for the proposed drugs were determined using the Discovery studio programme [23].

#### 2.19.2. Protein Preparation

The PDB was used to download the structures of the human IR (PDB id: 1IR3). After removing all the water molecules, the missing hydrogen atoms were supplied using the CHARMm force field’s Prepare protein module. As the experimental structure of GLUT-4 was not available, it was created using the AlphaFold method [24].

#### 2.19.3. Molecular Docking Procedure

Molecular docking investigations were conducted using the Discovery Studio module Ligand Fit. An active site is the portion of the receptor that is within 12 of the ligand’s geometric centroid. A total of 10 poses were formed during docking. The best poses were selected based on dock score values obtained after energy minimization. This utilized smart minimization and a molecule’s optimal orientation in the active site. The formula for calculating the dock score is as follows. A consensus scoring system was developed because a single docking score may not be enough to find active compounds. LigScore1, LigScore2, Jain, Piecewise Linear Potential (PLP1 and PLP2), and Mean Force Potential (PMF). The active compounds were chosen using a consensus scoring algorithm and their H-bond interaction with the receptor.

### 2.20. Molecular Simulation and Dynamics

#### 2.20.1. Molecular Dynamic Simulation for IR Complex

All-atom MD simulations were run for 100 ns on all the receptors in their free state (apo), as well as docked complexes at 300 K using the GROMOS 54A7 force field in the GROMACS simulation programme [25]. The apo and docked complexes were solvated in a cubic box (size 1.0 nm) and neutralized with sodium ions using the SPC water model. The PRODRG server was used to build an MD-based Ligand topology file for the docked complex [26]. With 1500 ps, the steepest descent approach was utilized to achieve energy minimization. The system temperature was initially fixed to 0 K and subsequently rose to 300 K over the equilibration phase. After that, an equilibration period of 100 ps, with constant volume, was achieved under periodic boundary conditions with a stable pressure of 1 bar. Graphs were generated using the MD simulation data using Xmgrace [27]. To analyze the stability of the simulation, the Root Mean Square Deviation and Root Mean Square Fluctuation values, as well as the solvent accessible surface area (SASA) and radius of gyration (Rg), were calculated using the GROMACS simulation software. By means of the Molecular Mechanics Poisson–Boltzmann Surface Area method and the g mmpbsa package in GROMACS 5.0.7 software, we obtained the overall binding free energy of the docked complex, free solvation energy (which includes the sum of polar and non-polar solvation energies), and potential energy, as well as a cumulative score of electrostatic and Van der Waals interactions for each docked complex conformational change up to 100 ns in duration.

#### 2.20.2. Molecular Dynamics Simulation for GLUT-4

Desmond module simulations of Schrodinger complexes employing all atom force fields were used to study the behavior of chosen chemicals with receptors in the molecular dynamics (MD) (OPLS-2005). The TIP4Psystem was chosen because the protein was naturally water soluble. The volume of water and the neutralizing components of 0.15 Na+Cl in the system were safely contained within an orthorhombic box [28]. An orthorhombic simulation box with a minimum 5 nanosec timescale was used to incorporate the proteins using the Desmond system builder facilities. As a result, NPT was chosen to represent the dynamic ensemble attributes atom count, pressure, and timeframe. As dynamic simulations were being performed in an even volume, it was imperative that the density and pressure were exact [29]. By measuring the box size after successful stabilization of pressure, it was possible to ensure the correct atom count (density of whole system). Nose-Hoover thermostats and the Martina-TobiasKlein barostat method were utilized to maintain the temperature level at 300 K during the whole dynamic simulation. An inner time step of 2.0 fs was used for bound interactions and non-bonded interactions within the short-range cut-off to integrate the equations of motion [30].

## 3. Results

### 3.1. Phytochemical Analysis of Ethanolic Extract of C. papaya

The phytochemical screening of the ethanolic extract of the leaves of *C. papaya* demonstrated the presence of tannins, saponins, alkaloids, flavonoids, glycosides, triterpenoids and phenols, as shown in Table 3. The qualitative analysis of the phytochemicals in the leaves of *C. papaya* also showed the presence of acid and proteins.

### 3.2. In Vitro Antioxidant Analysis

#### 3.2.1. DPPH Free Radical Scavenging Activity of *C. papaya*

**Figure 1:**
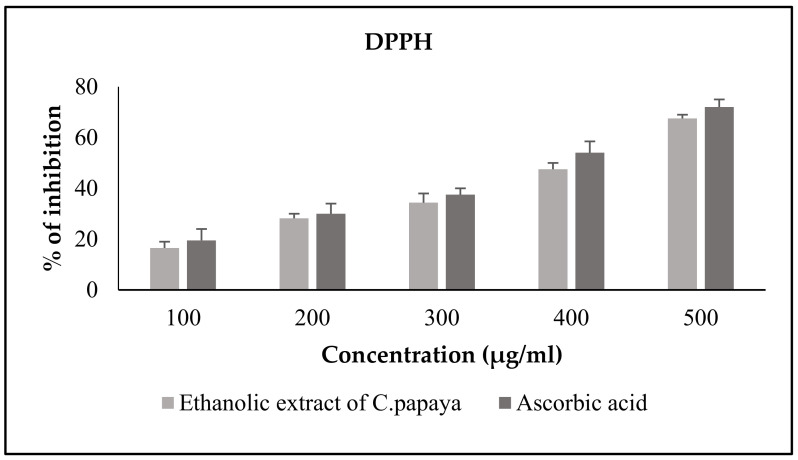
Effect of *C. papaya* ethanolic extract on DPPH radical scavenging activity. Each bar represents the mean ± SEM of five observations.

The DPPH free radical scavenging assay estimated the antioxidant potential of the *C. papaya* leaves with a sample concentration from 100 to 500 μL, in comparison to the standard ascorbic acid. The extract percentage of inhibition was significant (*p* < 0.05) in comparison to the standard percentage inhibition. Figure 1 represents the DPPH free radical scavenging activity of the ethanolic extract of *C. papaya* against the standard ascorbic acid.

#### 3.2.2. Nitric Oxide Radical Scavenging Activity of *C. papaya*

**Figure 2:**
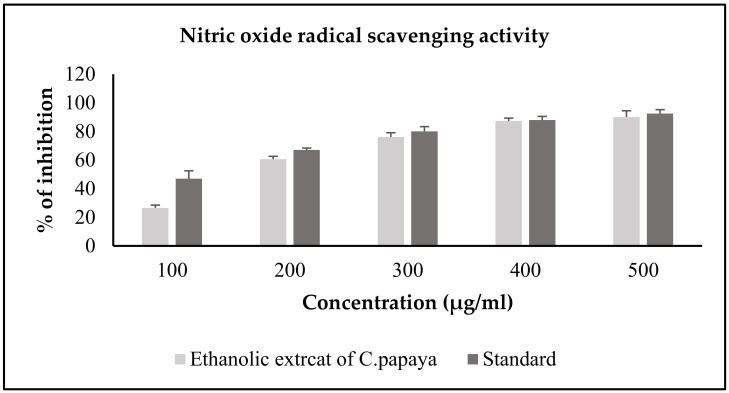
NO radical scavenging activity of the ethanolic extract of *C. papaya*. Each bar represents the mean ± SEM of five observations.

The sample concentration from 100 to 500 μg of *C. papaya* and ascorbic acid inhibited the nitric oxide radical scavenging in a dose dependent manner. The extract percentage inhibition in nitric oxide radical scavenging activity of *C. papaya* was more desirable than the standard percentage inhibition, and were 45.6%, 65.6%, 77.6%, 86.4% and 88% at the concentration of 100 μg, 200 μg, 300 μg, 400 μg and 500 μg, respectively, and was more desirable than the standard percentage inhibition. The nitric oxide radical scavenging activity of the ethanolic extract of *C. papaya* vs. ascorbic acid is shown in Figure 2.

#### 3.2.3. Super Oxide Radical Scavenging Activity of *C. papaya*

**Figure 3:**
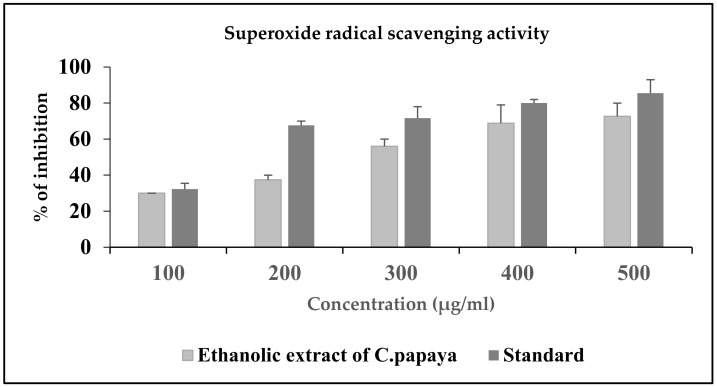
SO radical scavenging activity of the ethanolic extract of *C. papaya.* Each bar represents the mean ± SEM of five observations.

The *C. papaya* extract inhibited the superoxide radicals in a dose dependent pattern, as shown in Figure 3. The percentage inhibition was 29.9%, 34.7%, 52, 58.6% and 65.2% at the concentration of 100 μg, 200 μg, 300 μg, 400 μg and 500 μg, respectively. The standard ascorbic percentage inhibition was 35.5%, 65.2%, 65.2%, 78% and 78%, respectively, from a sample concentration of 100–500 μg. A concomitant increase in the superoxide radical scavenging activity of *C. papaya* was observed from 300 to 500 μg.

### 3.3. Effect of C. papaya on Fasting Blood Glucose

The diabetic rats displayed increased FBG levels when compared to the control rats (*n* = 8). Treatment with *C. papaya* reduced the FBG level, as was the case for the metformin treatment. Diab + *C. papaya* and Diab+metformin groups brought the levels of FBG down to a level close to the control group. These results are depicted in Table 4. The level of serum insulin was significantly high in the diabetic group. However, treatment with *C. papaya* significantly lowered the levels of serum insulin, and the effect was found to be equal to that of the metformin level.

### 3.4. Effect of C. papaya on Oral Glucose Tolerance (OGT)

**Figure 4:**
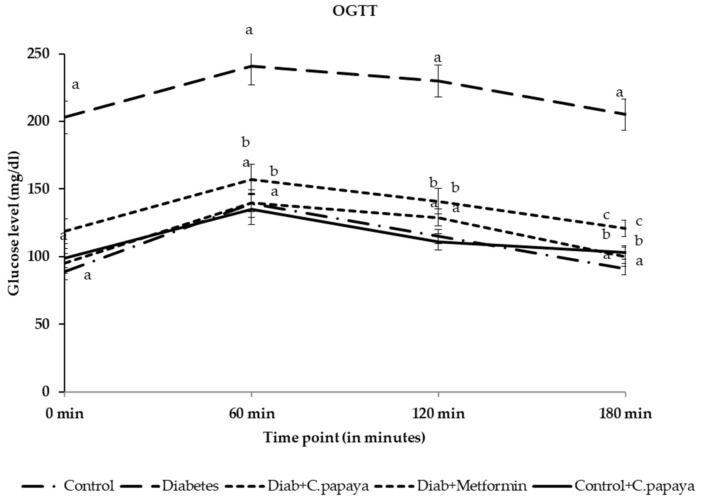
Effect of the ethanolic extract of *C. papaya* on oral glucose tolerance (OGT) in the control and experimental groups. Each line indicates the mean ± SEM of eight rats, with *p* < 0.05 indicating significant differences between the groups as: a—control; b—diabetes; and c—diabetic rats treated with the ethanolic extract of *C. papaya*.

An elevated blood glucose level was observed in the diabetic group after the glucose load and reached the maximum in one hour. After 2 h of glucose load, the 120 mg/dL range was not attained, thus indicating glucose intolerance. Administration with 600 mg of *C. papaya* enhanced glucose tolerance in a similar way as metformin (*n* = 8) (Figure 4).

### 3.5. Effect of C. papaya on Serum Insulin and HOMA-IR

A considerable increase was noted in the HOMA-IR of diabetic rats when compared to the control rats (*n* = 8). The treatment with *C. papaya* reinstated these levels close to the control group in Table 4. Administration with *C. papaya* to the control rats did not demonstrate any alteration compared to the control.

### 3.6. Effect of C. papaya on Lipid Profile

Serum triglycerides (TG), total cholesterol (TC), high density lipoproteins (HDL), and low density lipoproteins (LDL) were estimated in the five groups that each had eight rats in it. The diabetic rats displayed significantly high levels of serum triglycerides, total cholesterol and LDL, as well as a low level of HDL, as shown in Table 4. The treatment of *C. papaya* alleviated dyslipidemia in the Diab + *C. papaya* group, which was similar to the metformin treatment. The control rats treated with *C. papaya* did not show any changes.

### 3.7. Evaluation of C. papaya on Glycogen Level

**Figure 5:**
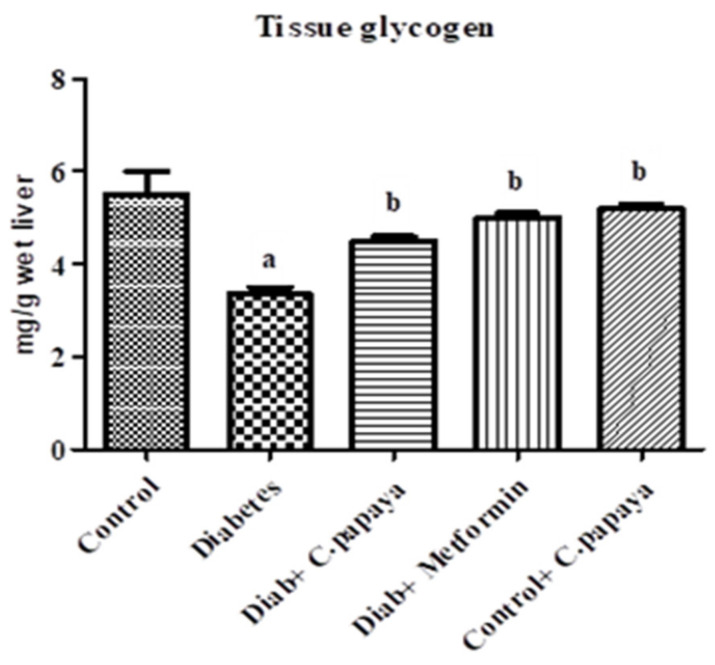
This figure Represents the outcome of the ethanolic extract of *C. papaya* on tissue glycogen contents in the control and diabetic rats. Each bar indicates the mean ± SEM of eight rats, with *p* < 0.05 indicating significant differences between the groups as: a—control and b—diabetes.

Figure 5 displays the concentration of glycogen in the control and experimental rats from the five groups that each had eight rats in it. The glycogen concentration was reduced in the gastrocnemius muscle of type 2 diabetic rats, as compared to the control group. Treatment with *C. papaya* partially reinstated this, which is similar to the metformin treatment.

### 3.8. Effect of C. papaya on Oxidation Stress Marker

**Figure 6:**
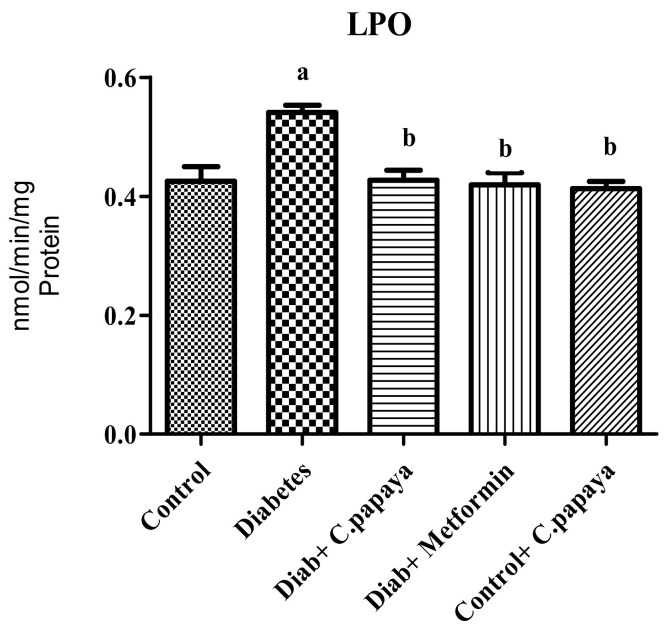
This figure represents the outcome of the ethanolic extract of *C. papaya* on the oxidative stress marker (LPO) in the skeletal muscles (gastrocnemius muscle) of control and diabetic rats. Each bar indicates the mean ± SEM of eight rats, with *p* < 0.05 indicating significant differences between the groups as: a—control and b—diabetes.

The oxidation stress marker, lipid peroxidation (LPO), was significantly high in the skeletal muscles of the diabetic group (*n* = 8). The LPO level was reduced in the skeletal muscle tissue (gastrocnemius muscle) after treatment of *C. papaya* close to the control group, as shown in Figure 6. A similar reduction in the level of the oxidation stress marker was observed in the metformin-administered group. There were no significant statistical changes in the gastrocnemius muscles of the control + *C. papaya* group.

### 3.9. Effect of C. papaya on Tissue Antioxidants Enzymes

**Figure 7:**
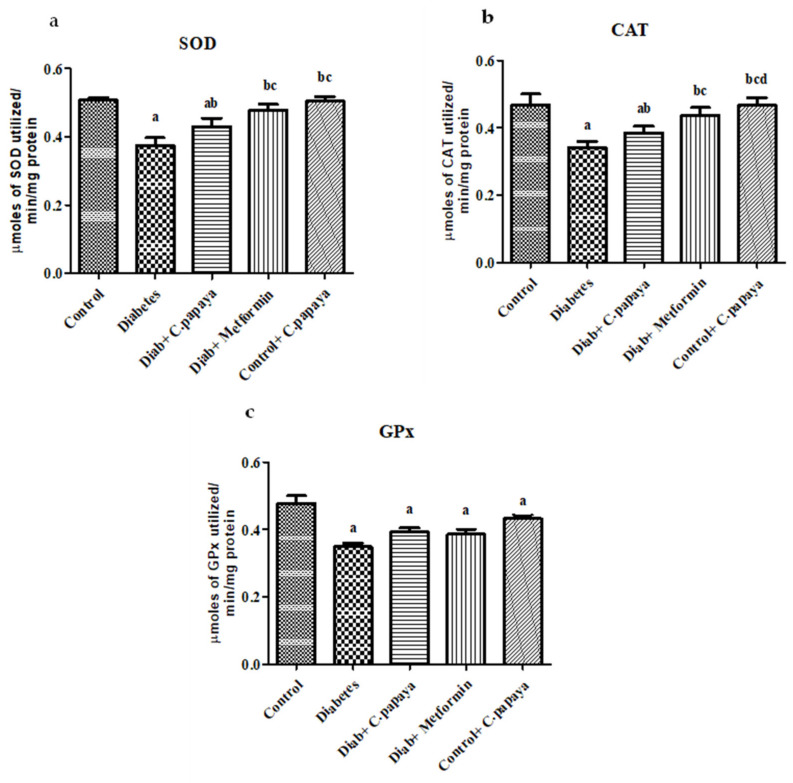
(**a**–**c**) Enzymatic antioxidant levels in the ethanolic extract of *C. papaya* in the control and diabetic rats. Each bar indicates the mean ± SEM of eight rats, with *p* < 0.05 indicating significant differences between the groups as: a—control; b—diabetes; c—diabetic rats treated with ethanolic extract of *C. papaya*; and d—diabetic rats treated with metformin.

Figure 7a–c shows the level of superoxide dismutase, catalase and glutathione peroxidase in the control and experimental rats of the five groups that each had eight rats in it. A significant decrease was noticed in the levels of superoxide dismutase (SOD), catalase (CAT) and glutathione peroxidase (GPx) in the skeletal muscle of the diabetic group when compared to the control group. These tissue levels of the antioxidants enzyme in the skeletal muscle of *C. papaya*-treated group were efficiently increased, as compared to the high fat diet, streptozotocin-induced diabetic group. The Diab and metformin groups also showed a significant increase in these levels. The control and *C. papaya* group rats did not show any alterations in these enzymatic antioxidant levels.

### 3.10. Effect of C. papaya mRNA Expression on IR and GLUT4

**Figure 8:**
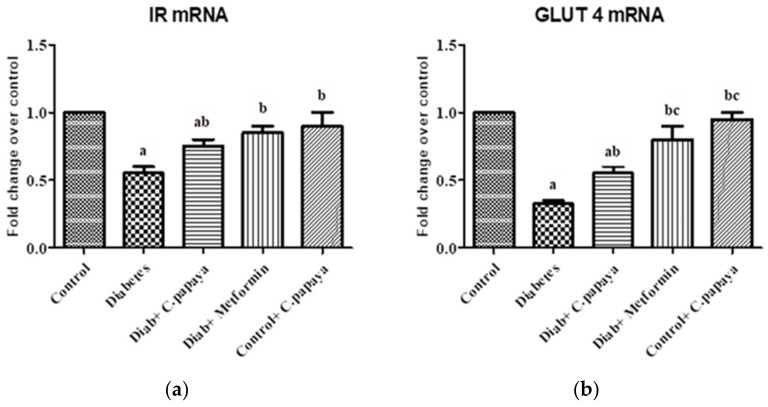
This figure represents the outcome of the ethanolic extract of *C. papaya* on IR (**a**) and GLUT4 (**b**) mRNA expression in the skeletal muscle of the control and diabetic rats. Each bar indicates the mean ± SEM of eight rats, with *p* < 0.05 indicating significant differences between the groups as: a—control; b—diabetes; c—diabetic rats administered with ethanolic extract of *C. papaya*.

The levels of IR mRNA in the control and experimental rats (*n* = 8) are shown in Figure 8a. The levels of IR proteins in the skeletal muscle were significantly decreased (*p* < 0.05) in the diabetic group. The treatment with *C. papaya* enhanced the IR gene expression in the gastrocnemius muscles in the Diab + *C. papaya* group, as was the case for the Diab + metformin group when compared to diabetic rats. These results infer the capability of *C. papaya* to escalate the insulin signaling in the skeletal muscles of diabetic animals. The trafficking of GLUT 4 in the plasma membrane and cytosol is a critical step for the muscle cell to potentiality use the blood sugar for glycolysis or glycogenesis. The levels of GLUT 4 in all five groups are shown in Figure 8b. The levels of GLUT 4 in the skeletal muscle of the diabetic group were significantly decreased (*p* < 0.05) when compared to the control group. Interestingly, *C. papaya* administration enhanced the gene levels of GLUT4 in the same way as for the metformin treatment.

### 3.11. Effect of C. papaya on the Histopathological Changes in the Skeletal Muscle

**Figure 9:**
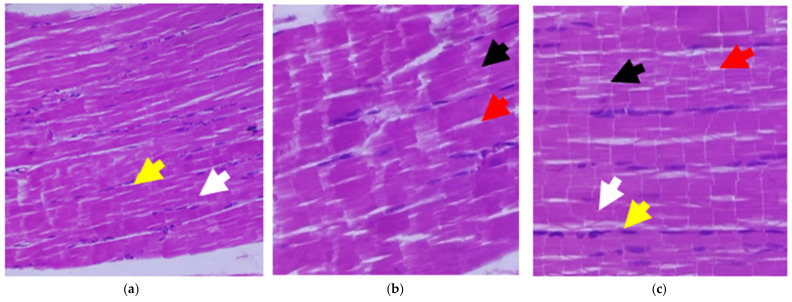

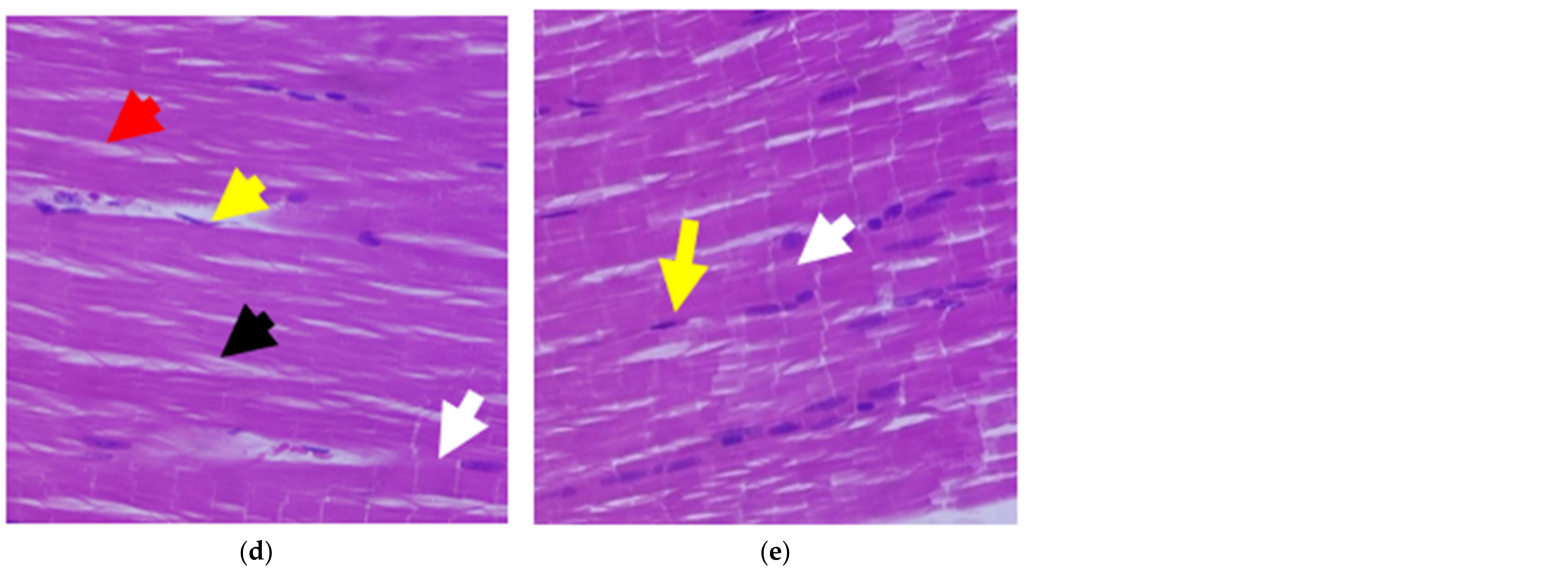
Effect of *C. papaya* on the histopathology of skeletal muscle tissue: (**a**) Control rats; (**b**) type-2 diabetic rats showed discontinuity in the muscle fiber’s (black arrow) wide space between the myofibrils (red arrow) when compared to the control; (**c**) type-2 diabetic rats treated with *C. papaya* (600 mg/kg b.wt) showed a restored architecture of skeletal muscle when compared to type-2 diabetic rats; (**d**) type-2 diabetic rats treated with metformin (50 mg/kg, b.wt) also restored the skeletal muscle structure to a level close to the normal control rats; and (**e**) control rats treated with *C. papaya* (600 mg/kg b.wt). Acidophilic myofibers arranged in bundles (white arrow) with a peripherally placed and flattened nucleus (yellow).

An histopathological examination of the skeletal muscles of the five groups in the experimental study (*n* = 8) is shown in Figure 9a–e. The induction of diabetes in the diabetic group caused an interruption between the skeletal muscle fibers and a reduction in the number of muscle fibers. The connective tissue space relatively increased in the diabetic group when matched with the control group. An abnormal positioning of nuclei, rather than being in the peripheral position, was noticed and was partly inserted into the muscle fibers. The differences in the nuclei sizes could also be seen in the diabetic group. These differences in the number of muscle fibers and the connective tissue space were considerably restored after treatment of *C. papaya* in the Diab + *C. papaya* group, which was similar to the metformin effect in the Diab + metformin group. The positioning of nuclei was gradually restored in the treatment groups of Diab + *C. papaya* and Diab + metformin. The control group treated with *C. papaya* showed a normal morphology.

### 3.12. In Silico Study

#### 3.12.1. Study of Pharmacokinetic Profiling

**Figure 10:**
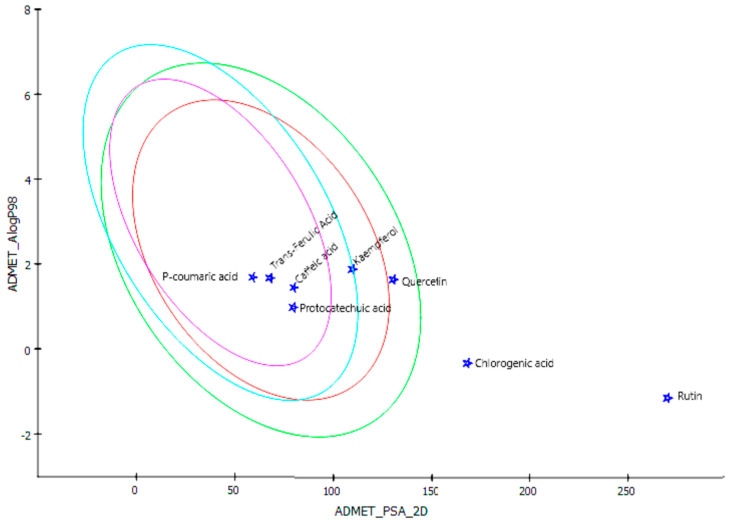
ADME analysis. Blue stars represent the proposed compounds of *C. papaya*.

The present work used in silico computational analysis to assess the physicochemical properties of compounds, in accordance with Lipinski’s rule of five. The ADMET Descriptors procedure estimates a range of ADMET-related attributes for a proposed substance using QSAR models. The depicted ellipses denote the locations that are likely to contain well-absorbed compounds: 95% of well-absorbed chemicals should lie inside the 95% ellipse, while 99% should fall within the 99% ellipse. It is important to remember that the position of a suggested compound does not always imply that it would be readily, moderately, or poorly absorbed. However, absorption tends to decrease significantly outside the 95 percent ellipse. The 95 percent (blue line) and 99 percent (magenta line) confidence ellipsoids in Figure 10 define these levels.

#### 3.12.2. Molecular Docking Based on Dock Score Proposed Natural Compounds and Selected Target Proteins (IR and GLUT4)

**Figure 11:**
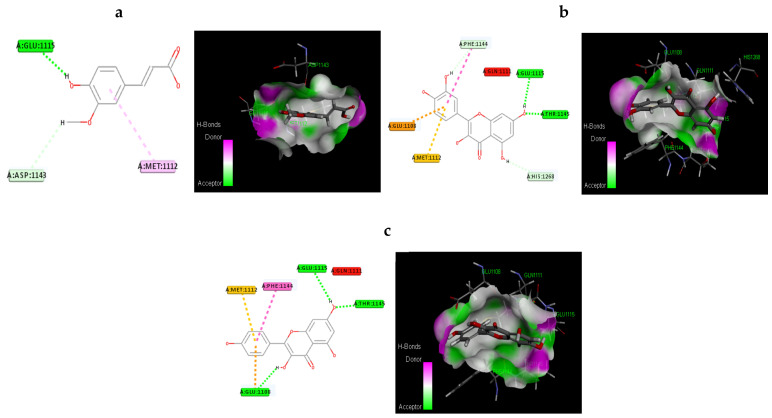
Molecular interaction of the best three compounds with IR: (**a**) Trans-Ferulic Acid, (**b**) Quercetin, and (**c**) Kaempferol.

**Figure 12:**
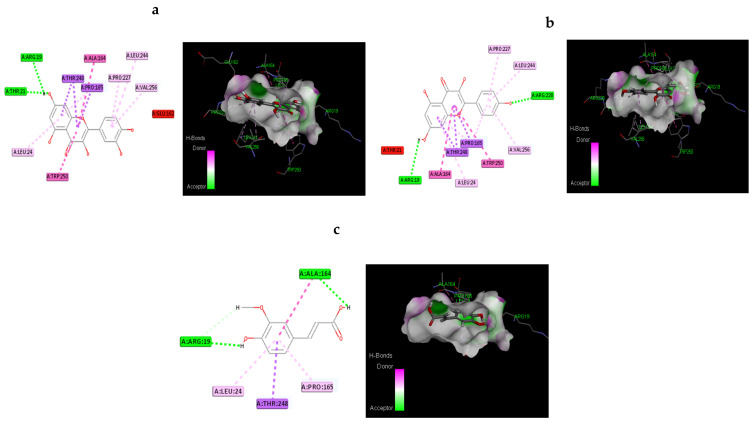
Molecular Interaction of the best three compounds with GLUT-4: (**a**) Quercetin, (**b**) Kaempferol, and (**c**) Trans-Ferulic Acid.

The proposed compounds were scrutinized, with the assistance of docking studies, using the Discovery Studio software and focused on their binding capabilities against IR and GLUT-4. The 3D structure of IR was obtained from the RCSB PDB and Alphafold-modelled structure of GLUT4 and was used in the present study. The results of this docking investigation revealed that selected compounds have a significant interaction with the target proteins. While docking into the active site, a ligand can experience van der Waals, hydrogen bonding, hydrophobic, and electrostatic interactions. According to the literature, binding energy has more influence in predicting the optimum binding mode than the number of contacts. The traditional H bond (HB) (which is more prevalent) and hydrophobic contacts are more effective than the others. Table 5 shows how distinct binding pocket amino acid residues associated with the phytocompounds. The binding energy and number of interactions clearly show that selected compounds have a higher affinity target protein. The best three compounds for each target protein were chosen based on the binding energy and interaction. Figure 11 and Figure 12 shows the interaction of the best compounds with each target proteins.

#### 3.12.3. Molecular Dynamic Simulation of IR

**Figure 13:**
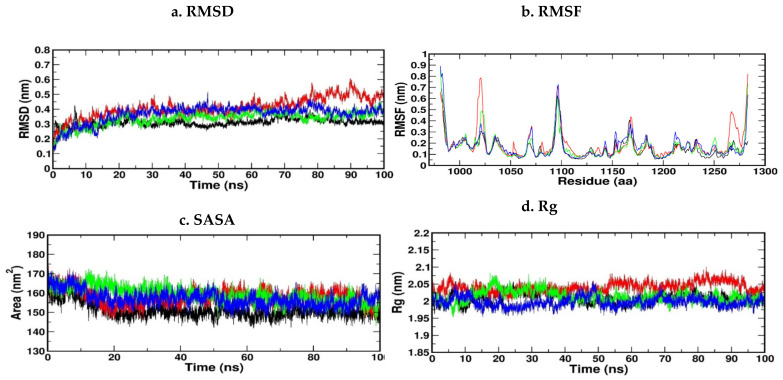
(**a**–**d**) RMSD, RMSF, SASA and RG of the IR protein with the top three compounds. Kaempferol shown in red, Quercitin (green), and Trans-Ferulic Acid (blue).

The top three compounds for the IR protein exhibited better binding energy. Thus, three docked complexes were suggested for MD simulation studies. Figure 13a shows the RMSD of the c-alpha atoms of IR, which showed that the hypothesized compounds–receptor docked complexes were initially stable but fluctuated somewhat after 10 ns. Throughout the remainder of the simulation periods, these complexes maintained a consistent RMSD profile. Additionally, at 35 ns, the proposed compounds and the receptor docked complex exhibited a comparable RMSD, which increased slightly after that, but all the complexes did not over-fluctuate, thus demonstrating a rigid conformation. Interestingly, none of the three complexes revealed RMSD characteristics that were more than 0.5 nm, thus indicating that the drug complex’s stability. High RMSF values suggest that MDS is more flexible. Figure 13b shows a plot of RMSF values against all the residues. The RMSF graph shows that all the complexes fluctuated less than 0.5 nm, while the docked complexes fluctuated more than 0.5 nm in the 400–440 residue position. In the initial phase of MDS, all the docked complexes proposed exhibited instability. The RMSF profile illustrates the flexibility among amino acid residues. Figure 13d also shows that the compounds and receptor complex had a comparable Rg profile from 0 to 20 ns, then a decreasing Rg descriptor, indicating the complex’s constricted packaging system, as the Rg of the drug complex reflects the protein’s compact nature. Furthermore, the compounds and the receptor complex displayed an abridged Rg profile from 0 to 20 ns and a greater Rg spike from 25 to 45 ns, indicating loose system packing. When compared to compound 1 and the compound receptor docked complexes, the compound 3 receptor docked complex displayed a moderate Rg profile, indicating that it was less mobile. Furthermore, the complex’s solvent-accessible surface area was examined using simulated trajectories to determine the volume change of the complexes over time. The SASA profile of the compounds and receptor complex was lower in the beginning, but raised later, indicating that the protein surface area expanded. As a result, during the remainder of the simulation trajectory, this complex’s volume was significantly reduced. Interestingly, the compounds and receptor docked complexes displayed an SASA trend that was higher, despite minor changes ranging between 35–40 ns (Figure 13c).

#### 3.12.4. Molecular Dynamics of GLUT-4

**Figure 14:**
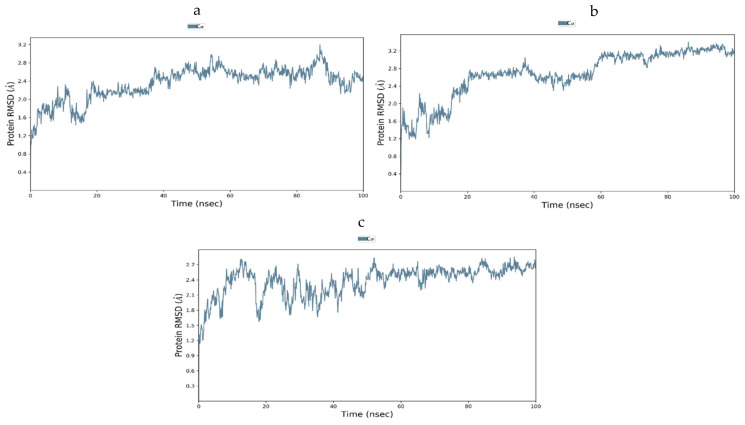
(**a**–**c**) RMSD plots for GLUT-4 with selected compounds: (**a**) Quercitin, (**b**) Kaempferol, and (**c**) Trans-Ferulic Acid.

**Figure 15:**
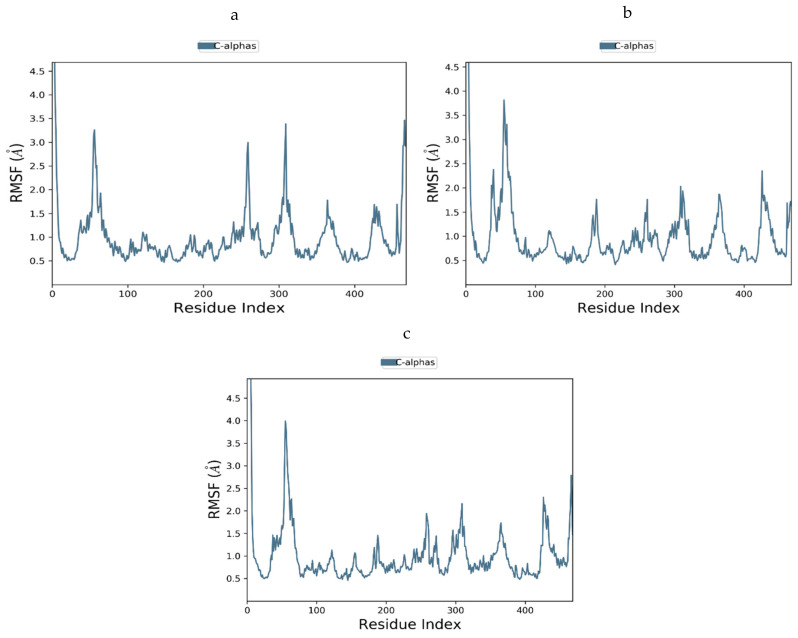
(**a**–**c**) RMSF plots for GLUT-4 and with selected compounds: (**a**) Quercitin, (**b**) Kaempferol, and (**c**) Trans-Ferulic Acid.

The steadiness of the GLUT-4-ligand complexes was evaluated using RMSD and RMSF. Figure 14a–c shows the RMSD graphs for the three complexes. There was very little fluctuation in the RMSF plots of docked complexes for the 1000 ns MD simulation paths, at least throughout the simulation period, as shown in Figure 15a–c. As can be seen, the compounds used were quite stable in the presence of the protein structure. Furthermore, no compounds were separated from the system throughout the dynamics, thus proving that the MD investigation greatly supported the projected binding positions. The outcomes of the dynamics simulation demonstrated that all the chemicals exhibited the certain state with the target protein. It did not move much because it was stuck in the same spots. There is now evidence that the chemicals and GLUT-4 protein interaction that we simulated has reached equilibrium, and that these are the best inhibitors of GLUT-4.

## 4. Discussion

The universal rate of the upsurge of diabetes has shown an alarmingly upward trend in recent decades and it will soar to greater heights in the next few decades worldwide. The varying interruptions in insulin secretion, insulin action, insulin resistance, glucose production and uptake could emerge into the status of an illness. Additionally, hormonal interactions and various stress factors could pave the way for this disorder [31]. One of the major causes of type 2 diabetes is peripheral insulin resistance, which is a crucial defect that is associated with obesity and a metabolic disorder. A diminished insulin-incited glucose uptake in the skeletal muscle can result in insulin resistance due to defective insulin signaling and multifarious, post-receptor intracellular faults with defective glucose transport, glucose phosphorylation, and diminished glucose oxidation and glycogen synthesis [32]. These multifactorial causes mean that the management of diabetes is a great medical encounter [31]. In this study, we focused on evaluating the anti-diabetic role of *C. papaya* high fat diet, streptozotocin-induced T2DM, and *C. papaya* can enhance the insulin sensitivity in the skeletal muscles of diabetic rats. Additionally, the bioactive compounds of *C. papaya* were scrutinized by molecular docking and dynamics simulation studies to identify the best bio-compounds against the IR and GLUT4.

*C. papaya* is lauded for its curative properties due to its antioxidative properties and every part of the fruit, roots, leaves, and seeds have been utilized as a folklore treatment of hyperglycemia [33]. Previous literature has recorded the presence of bioactive compounds such as alkaloid, flavonoid, tannin, saponin, glycoside, and triterpenoids in their screening of phytochemicals of the ethanolic extract of leaves of *C. papaya* [34,35,36]. In our phytochemical investigation of the ethanolic extract of *C. papaya* leaves, phytocompounds, such as triterpenoids, flavonoids, phenols, tannins, alkaloids, and glycosides, were found. In addition to these phytocompounds, the presence of acid and proteins were also shown in our analysis. The presence of different bioactive compounds can be known by phytochemical screening, but the pharmacological actions cannot be solely resolute. The literature shows that the hypoglycemic and hypolipidemic outcome could be due to the flavonoids, alkaloids, steroids and quinones [34]. Considerable literature has shown that hyper glycaemia induces oxidative stress by triggering the production of ROS and debilitates the antioxidant aegis through numerous mechanisms, such as eliciting protein glycation, which ultimately promotes the production of advanced glycation end products (AGEs) and increases free radicals. These excessive free radicals can lead to metabolic complications. Thus, the role of antioxidants alleviates free radicals and avoids the complications [33]. Earlier literature has centered on the DPPH scavenging activity of different parts of *C. papaya,* such as leaves, seeds, flowers and fruits [37,38,39,40]. The DPPH assay of fluorescent carbon dots prepared from the leaves of *C. papaya* proved an efficacy of about 80% radical scavenging activity [41]. The DPPH radical scavenging nature of the aqueous extract of the leaves of *C. papaya* denoted the IC_50_ value of 198 µg/mL at a 500 µL sample concentration, and the extraction percentage inhibition was 72.92 ± 1.45 [42].

In our study, we assessed the DPPH radical scavenging property of the ethanolic extract of *C. papaya* against ascorbic acid, which is the standard antioxidant. The extract percentage inhibition was comparatively better than the standard percentage inhibition, and at 500 µL, our extract percentage inhibition was 90.9, which was far better than the extraction percentage inhibition reported previously by Srikanth et al. [42]. Nitric oxide is an unsteady free radical entail with many diseases in the biological processes. Overproduction of nitric oxide free radical leads to many pathogenic conditions and reports have shown that plant extracts contain nitric oxide radical scavengers that thrive with oxygen, leading to reduced nitrite ion production [43]. In a previous study, the methanolic extract of *C. papaya* leaves had a percentage inhibition of 72.91 at 60 µg/mL [44]. In our assay of nitric oxide radical scavenging, a dose dependent inhibition was found from 100 to 500 µL of the sample concentration against ascorbic acid and the percentage inhibition at 500 µL was 88. These results show a better scavenging activity of the ethanolic extract of *C. papaya* than the previous studies. Superoxide radicals are very destructive to cellular components, as a forerunner of the reactive oxygen species, aids tissue damage and is harmful to the biological system. An earlier study of the superoxide scavenging activity of *C. papaya* seeds suggested that they could avoid oxidative damage to the cells [38]. A concentration dependent inhibition of superoxide scavenging activity, from 11.86% to 100%, was reported with an increased concentration of the methanolic extract of *C. papaya* leaves [45]. In our study, we found a dose-dependent inhibition of scavenging superoxide radicals of the ethanolic extract of *C. papaya* leaves with a percentage inhibition of 77.8. The leaves of *C. papaya* displayed strong free radical scavenging activity due to the phytochemicals present in them and they can remove the free radicals that increase due to T2DM progression.

In the present study, high fat diet, streptozotocin-induced T2DM caused obliteration in the insulin action, thus affecting the plasma glucose regulation and resulting in hyperglycemia. A diminution in metabolic response to insulin in the target cells, triggering a hinderance to decrease the high blood glucose levels by circulating insulin, causes insulin resistance. Insulin resistance boosts insulin secretion, but weakens the metabolism in the skeletal muscle, liver and adipose tissue, thus causing peripheral insulin resistance, and these result, due to the excessive output of reactive oxidative species, in faulty glucose and fatty acid oxidation, ER stress and mitochondrial impairment [46,47,48]. The diabetic group in our study displayed a significant increase in FBG, OGTT and serum insulin. Hyperinsulinemia can lead to disorders, such as obesity, hypertension, hyperlipidemia, and coronary artery disease [47]. People lose their initial insulin secretory sensitivity to glucose and may even generate huge quantities of proinsulin [49]. The elevated levels of FBG and serum insulin are attributable to insulin resistance-caused hyperglycemia. The blood glucose level of type 2 diabetic rats in OGTT progressively elevated and attained its peak value at one hour, but was not marked within the normal array after two hours of glucose load, featuring glucose intolerance in the diabetic rats. Furthermore, rats treated with *C. papaya* appreciably reduced their fasting blood glucose, OGTT and serum insulin after an enhancement in insulin sensitivity and IR-facilitated glucose uptake and oxidation by *C. papaya*. The gradation of insulin resistance was determined by HOMA-IR and was calculated from the fasting plasma glucose and insulin levels. The HOMA-IR was elevated in the diabetic rats in our study and treatment with *C. papaya* was able to bring the HOMA-IR index back to close to normalcy due to the capability of *C. papaya* to restore insulin signaling, which is same as for metformin.

Hyperlipidemia, another major factor in diabetic pathogenesis and related consequences, is conspicuous in diabetic rats. In the present study, the diabetic serum lipid profile showed a rise in serum triglycerides (TG), total cholesterol (TC) and LDL-c due to excessive synthesis of lipids in liver, leading to a surge in the levels of plasma lipid [50] and dwindling HDL cholesterol levels. An impairment of LDL receptors to clear plasma LDL-c may have caused the high levels of LDL and VLDL in diabetic animals. An upsurge in the hepatic cholesterol level represses the transcription activity of the LDL receptor genes and plasma LDL gradually amasses. Thus, the LDL synthesis and its clearance from the circulation plays a major role in determining the LDL plasma concentration [51]. A surplus number of metabolites of TG and FFA can restrict the stimulation of phosphatidylinositol-3-kinase (PI3K)/Akt, thus causing a dip in the downstream signaling actions of insulin and resulting in insulin resistance [52]. Furthermore, rats treated with *C. papaya* had appreciably reduced serum lipid profiles, but elevated HDL cholesterol levels caused by inducing a cholesterol transport process, indicating its hypocholesterolemia, which is in support of previous studies [53].

Reactive oxygen species (ROS) generation from dyslipidemia can damage membranes, inducing lipid peroxidation and leading to systemic oxidative stress [54]. In our study, the levels of lipid peroxidation were increased in diabetic rats, which could be due to increased blood glucose levels that cause ROS production, either by non-enzymatic glycation of proteins or auto-oxidation [55]. However, the administration of *C. papaya* lowered the levels of LPO in the skeletal muscle of diabetic rats. *C. papaya*, which possess an anti-oxidant potential, can alleviate overproduced ROS. Ezymatic antioxidants, such as CAT, SOD, and GPx, eradicates the free radicals from cells. These antioxidant levels were diminished in diabetic rats. The administration of *C. papaya* considerably increased antioxidant enzyme levels, which greatly reduced the ROS and avoided lipid peroxidation in the skeletal muscle of diabetic rodents. This was as effective as metformin. These findings were quite similar to those obtained by Raffaelli F et al. [56]. That is, *C. papaya* upregulated the SOD level and mitigated lipid peroxidation by displaying a defensive result towards diabetic complications, such as atherosclerotic plaque formation.

The tissue glycogen concentration was considerably lowered in the skeletal muscle of *C. papaya* treated diabetic rodents. The shortcomings in glycogenesis results in a weakened Akt phosphorylation at Thr308, which is a primary factor required for the initiation of glycogen synthase [52,57]. On treatment with *C. papaya*, the content of glycogen in Group 3 rats were brought back to normal levels, which were as good as metformin.

Diminished, insulin-stimulated glucose uptake in skeletal muscle is an indication of insulin resistance, due to defective insulin signaling and a number of intracellular post-receptor abnormalities, including reduced glucose oxidation and glycogen formation and impaired glucose transport. The insulin receptor is part of the receptor tyrosine kinase (RTK) family, which also includes the receptors for other growth factors, in addition to insulin and insulin-like growth factors [58]. Insulin binds with the insulin receptor (IR), followed by the activation of the tyrosine kinase receptor, in succession with phosphorylates to activate IRS proteins. Phosphorylated IRS provides docking sites for phosphatidylinositol 3 kinase (PI3K) that stimulates Akt/protein kinase B, causing an enhancement in the translocation of intracellular GLUT4 to the plasma membrane. The IRS/PI3K/Akt pathway triggers glucose utilization in the cells of the skeletal muscle. This is defective for the skeletal muscle in conditions such as obesity, hypertension, and type-2 diabetes mellitus [59,60]. In the present study, the level of IR protein was reduced in the skeletal muscle of the high fat diet, streptozotocin-induced diabetic rats and could be due to the elevated levels of free fatty acids that dwindle the IR gene expression of the cells [52]. A significant drop in IR protein expression and the skeletal muscle of diabetic rats in our experiment was noted. Administration of *C. papaya* flaunted an upsurge in IR protein levels owing to the hypolipidemic property of *C. papaya.*

A significant drop in the IR protein expression and skeletal muscle of diabetic rats in our experiment was noted. Administration of *C. papaya* flaunted an upsurge in IR protein levels owing to the hypolipidemic property of *C. papaya.* The GLUT4 transporter is responsible for glucose absorption in skeletal muscle. GLUT4 vesicles switch from the cytoplasm to the plasma membrane when insulin binds to its receptor and mediates glucose absorption by cells. A reduced shuttle of GLUT4-source insulin resistance in type 2 diabetes mellitus was noted [57,61]. In the current study, diabetic rats presented considerable diminution in the expression of GLUT4 in the plasma membrane, as well as cytosol, which may be due to uncontrolled FFA levels, which in turn lessen the expression and shifting of GLUT4 to the plasma membrane [52,62]. However *C. papaya* treatment increased the GLUT4 levels in the plasma membrane and cytosol, as *C. papaya* facilitated an upsurge in IR. This depicts the molecular mechanism of antidiabetic capability of *C. papaya.*

The muscle fibers in the skeletal muscle of the diabetic rats were reduced in number, shrunken, distorted with nuclei paucity and had increased connective tissue gaps between muscle fibers. These were the histopathological changes that were reformed through *C. papaya* treatment. These results were similar to those obtained by Samir et al. [63]. These developments, which occurred with *C. papaya* treatment, might be due to the prevention of oxidative stress and improved protein expression in the peripheral insulin targets.

The discovery of active molecules from natural sources have risen to prominence as a critical component of drug discovery. The present work used docking and dynamic studies to anticipate the compound’s antidiabetic efficacy at the target level. However, for the binding affinity analysis, the scores were transferred from the table browser view of the Discovery studio for the top-ranked docked complex. We have identified and proposed antidiabetic peptides for oral administration, employing an in silico method in our work. The ligands were thoroughly assessed via Lipinski’s rule of five and ADMET profiling, which dynamically reinforced the antidiabetic effects. Kaempferol, quercitin and transferulic acid were the top three ligands with the greatest hit rate against the protein targets of IR and GLUT4, which may act as adjuvant drugs to tackle diabetes with lesser or no complications. These bioactive compounds should be validated by wet-lab investigations.

## 5. Conclusions

*C. papaya* has the potential to upgrade the blood sugar levels and lipid profile in both the plasma and tissue of diabetic rats. *C. papaya* reinstated the glycaemic effect in the diabetic skeletal muscle by accelerating the IR and GLUT4 levels. The novelty of this study is that as we are the first to account for the conceivable role of *C. papaya* on insulin signaling molecules like IR and Glut4 in a high fat diet, streptozotocin-type 2DM model and also proposed, via molecular docking and dynamics, the top ligands, namely kaempferol, quercitin and transferulic acid of *C. papaya,* which docked well with IR and Glut4. The potential effect of *C. papaya* can be exemplified by studying the expression of other insulin signaling molecules like IRS-1 and Akt. The results of in-silico studies also supported our experimental studies. Therefore, it is evident that *C. papaya* may be a reassuring drug for the type DM management.

## Figures and Tables

**Table 1:** List of rat primers used.

S.No	Gene Name	Primer Sequence	Reference
1	Rat βactin	Sense primer: 5′- AAG TCC CTC ACC CTC CCA AAA G-3′ Anti-sense primer: 5′- AAG CAA TGC TGT CAC CTT CCC-3′	[18]
2	Glut-4	Sense primer: 5′- GGG CTG TGA GTG AGT GCT TTC-3′ Anti-sense primer: 5′- CAG CGA GGC AAG GCT AGA-3′	[19]
3	IR	Sense primer: 5′- GCC ATC CCG AAA GCG AAG ATC-3’ Anti-sense primer: 5′- TCT GGG TCC TGA TTG CAT-3’	[20]

**Table 3:** Qualitative phytochemical screening of the ethanolic extract of *C. papaya*.

S.No	Phytoconstituents	Ethanolic Extract of *C. papaya*
1	Phytosterols	−
2	Triterpenoids	+
3	Flavonoids	+
4	Phenols	+
5	Tannins	+
6	Alkaloids	+
7	Saponin	+
8	Acid	+
9	Proteins	+
10	Carbohydrates	−
11	Glycosides	+

**Table 4:** Effect of the ethanolic extract of *C. papaya* on body weight, fasting blood glucose, serum insulin, HOMA-IR and lipid profile in the control and experimental groups. a—control; b—diabetes; c—diabetic rats administered with ethanolic extract of *C. papaya*; d—diabetic rats treated with metformin.

Grouping	Body Weight (in g)		FBG (mg/dL)	Serum Insulin (µIU/dL)	HOMA-IR	Lipid Profile (mg/dL)			
	O Day	45th Day				TG	TC	LDL-c	HDL-c
Control	183 ± 5.2	210 ± 6.7	111 ± 9.5	0.275 ± 0.025	3.7 ± 0.39	109 ± 9	96 ± 6.5	92 ± 7.5	121 ± 10.5
Diabetes	189 ± 6.9	310 ± 9.3 ^a^	213 ± 14 ^a^	0.470 ± 0.020 ^a^	8.9 ± 0.71 ^a^	246 ± 16.5 ^a^	264 ± 14 ^a^	194 ± 5.5 ^a^	46 ± 8.5 ^a^
Diab + *C. papaya*	170 ± 4.3	235 ± 8.0 ^ab^	155 ± 13 ^ab^	0.349 ± 0.020 ^b^	5.3 ± 0.61 ^ab^	137 ± 6.5 ^ab^	135 ± 9.5 ^b^	127 ± 7.5 ^ab^	92 ± 10 ^b^
Diab + Metformin	194 ± 6.9	222 ± 6.9 ^abc^	130 ± 9 ^abc^	0.364 ± 0.014 ^b^	4.6 ± 0.41 ^abc^	117 ± 5.5 ^bc^	109 ± 8 ^b^	109 ± 12.5 ^b^	106 ± 6.5 ^b^
Control + *C. papaya*	179 ± 4.9	201 ± 9.2 ^bcd^	117 ± 8.5 ^bcd^	0.298 ± 0.018 ^b^	3.9 ± 0.39 ^bcd^	107 ± 8.5 ^bc^	104 ± 6 ^b^	101 ± 12 ^bc^	135 ± 5.5 ^b^

**Table 5:** Binding affinity assessment based on the dock score proposed natural compounds and selected target proteins (IR and GLUT4).

S.No	Compound Name	Lig Score1_Dreiding	Lig Score2_Dreiding	PLP 1	PLP 2	JAIN	PMF	Dock Score
IR								
1	Trans-Ferulic Acid	2.67	3.78	72.66	63.58	1.6	61.46	67.774
2	Quercetin	3.28	3.41	86.87	91.19	1.77	104.6	81.475
3	Kaempferol	2.87	3.53	81.13	85.28	1.99	99.7	76.777
4	Rutin	3.25	1.88	122.11	113.91	2.37	142.19	114.771
5	p-coumaric acid	No interaction						
6	Chlorogenic acid	No interaction						
7	Protocatechuic acid	No interaction						
8	Caffeic acid	2.78	3.63	62.17	54.35	0.55	57.25	59.454
GLUT4								
1	Trans-Ferulic Acid	3.18	4.48	78.29	77.17	−0.77	32.06	74.475
2	Quercetin	2.69	4.3	100.91	104.57	1.23	56.06	94.607
3	Kaempferol	3.24	4.28	98.43	102.65	0.81	54.23	92.185
4	Rutin	4.84	3.17	131.02	143.21	3.98	116.67	116.012
5	p-coumaric acid	2.74	4.35	68.22	64.13	−0.47	32.16	65.275
6	Chlorogenic acid	3.42	4.48	117.78	120.88	−0.31	82.04	108.434
7	Protocatechuic acid	1.14	3.46	69.08	73.26	−0.92	30.13	64.194
8	Caffeic acid	2.87	4.53	76.01	77.06	−0.91	34.51	72.9

## Data Availability

The data presented in this study are available in this article.
